# Saccade Generation by the Frontal Eye Fields in Rhesus Monkeys Is Separable from Visual Detection and Bottom-Up Attention Shift

**DOI:** 10.1371/journal.pone.0039886

**Published:** 2012-06-27

**Authors:** Kyoung-Min Lee, Kyung-Ha Ahn, Edward L. Keller

**Affiliations:** 1 Department of Neurology, Seoul National University, Seoul, Republic of Korea; 2 The Smith-Kettlewell Eye Research Institute, San Francisco, California, United States of America; University of Alberta, Canada

## Abstract

The frontal eye fields (FEF), originally identified as an oculomotor cortex, have also been implicated in perceptual functions, such as constructing a visual saliency map and shifting visual attention. Further dissecting the area’s role in the transformation from visual input to oculomotor command has been difficult because of spatial confounding between stimuli and responses and consequently between intermediate cognitive processes, such as attention shift and saccade preparation. Here we developed two tasks in which the visual stimulus and the saccade response were dissociated in space (the extended memory-guided saccade task), and bottom-up attention shift and saccade target selection were independent (the four-alternative delayed saccade task). Reversible inactivation of the FEF in rhesus monkeys disrupted, as expected, contralateral memory-guided saccades, but visual detection was demonstrated to be intact at the same field. Moreover, saccade behavior was impaired when a bottom-up shift of attention was not a prerequisite for saccade target selection, indicating that the inactivation effect was independent of the previously reported dysfunctions in bottom-up attention control. These findings underscore the motor aspect of the area’s functions, especially in situations where saccades are generated by internal cognitive processes, including visual short-term memory and long-term associative memory.

## Introduction

Initially recognized as an oculomotor area [1], the FEF have also been shown to play a role in much broader behavioral contexts, such as target selection [2]–[4], motor preparation [5], internal monitoring [6], adjustment of on-going saccades [7], inhibition of reflexive saccades [8], and shift of spatial attention [9]. Recently, numerous studies have implicated its function even in perceptual domains, such as in building and maintaining a visual saliency map [10], visual prediction [11], working memory of the visual world [12], and shifting visual attention [13]–[15]. Indeed, a majority of FEF neurons exhibit phasic or sustained visual responses with or without motor activity [16].

However, whether the FEF is causally involved in these various visual and cognitive functions remains unclear. While reversible or permanent lesions of the FEF lead to demonstrable errors in visuo-oculomotor tasks [17]–[21], it has been difficult to specify whether the lesions impinge upon visual or oculomotor functions, because the visual target and the saccade response were spatially confounded in the tasks employed in the investigations. Furthermore, visual attention was co-localized with saccade planning in most previous task paradigms: A transient visual change occurred at a position in the peripheral fields, triggering a shift of bottom-up attention as well as a saccadic movement of the eyes to the same position. Recently, a few studies have provided evidence that the oculomotor and the attentional roles by the FEF are in fact separable: FEF inactivation disrupted covert visual search in the absence of eye movements [22]. Shifts of gaze and shifts of attention may be carried out by different cell types [23] and different dopaminergic receptors [24] in this cortical area.

Here, using reversible inactivation techniques and two novel behavioral tasks, we aimed at dissecting cognitive processes underlying the visuo-oculomotor transformation often ascribed to this area. The results suggested a distinction between visual detection and saccade generation in FEF functions, as well as between bottom-up attention shift and saccade target selection by the cortex.

## Materials and Methods

### Ethics Statement

All experimental procedures were approved by the Seoul National University Hospital Animal Care and Use Committee (IACUC No: 09–0166, Project Title: Neural mechanisms of saccade choice in primate frontal cortex).

### Subjects and Surgical Preparation

Two adult female rhesus monkeys (*Macaca mulatta,* M9 and M10) weighing between 4 and 5 kg were used. A head-restraint post and recording cylinders were implanted under isoflurane anesthesia and sterile surgical conditions. The recording cylinders (20 mm, internal diameter) were positioned over craniotomies centered on the right arcuate sulcus in all animals.

### Procedures to Minimize Animal Discomfort, Distress, Pain and Injury

Three situations existed in which a monkey might experience discomfort, distress and/or pain in our experimental protocols: a) survival surgery; b) restraint for handling or routine testing and c) training and experimental recording sessions. The following steps were taken to ameliorate animal suffering in each situation. **a) Survival surgery.** The purpose of the surgical procedures was to implant recording chambers and a head restraint device for neurophysiological experiments. All surgeries were carried out in the animal surgical suite at the Primate Center of Seoul National University Hospital. Animals were prepared with sterile, anesthetic surgical procedures. A licensed veterinarian was present throughout the surgical procedures and the recovery period for anesthetic induction and for monitoring and recording all measured physiological variables. Animals were allowed free access to water but no food the night prior to scheduled surgery. One hour before the surgery the animal was given atropine sulfate (0.08 mg/kg, I.M.) to prevent excessive salivation during the surgery. One-half hour later it was sedated with zoletil chloride (10 mg/kg, I.M.), intubated, and placed under Isofluorane anesthesia. A saline drip was maintained through an intravenous catheter placed into a leg vein. Throughout the surgery, core body temperature, heart rate, blood pressure, oxygen saturation and respiratory rate was continuously monitored. The animal was returned to its home cage after waking from the anesthesia and allowed to recover fully from the effects of surgery before behavioral training started. During the period of post-surgical recovery the animal was monitored closely and given injections of an analgesic agent (meloxicam 0.4 mg/kg I.M.) and antibiotics (cephazolin, 25/mg/kg) in consultation with the veterinarian for 3 days post-op. **b) Restraint for handling or routine testing.** Restraint for certain procedures, such as physical examination or blood sampling for health check, was accomplished with zoletil chloride (10 mg/kg, I.M.). **c) Training and experimental recording sessions.** After recovery from the surgical procedure the animal was trained to be held by the arms and moved into a large plastic primate chair. This was done by supplying the animal with rewards of fruit and juice. The chair had a perch with an adjustable height for each animal’s comfort. Wastes fell into a collection pan below the animal, and thus, did not cause the animal discomfort. The animals were trained by the delivery of water or fruit juice rewards in daily sessions during which time they received their entire liquid intake in the experimental apparatus. When the animal was fully trained the experiments began. During the experimental sessions the animal’s head was painlessly restrained through the use of the implanted head post which mated to a vertical rod attached to the primate chair. The animals did not show any sign of discomfort by the head restraint device: They continued to train steadily for the period of time that they were in restraint and often fell asleep as they sat in the darkened room between blocks of trials.

### Behavioral Tasks

#### A) The extended memory-guided saccade task (Figure 1a)

A trial began with illumination of a central fixation spot (a gray disc, 0.5 degree in diameter). Visual stimuli were presented 400 ms after the animal acquired fixation at the spot. When a single stimulus was briefly (200 ms) shown on either side, as in the traditional version of the task, the animal was required to remember the location, wait through a delay period ranging between 800 and 1200 ms, and make a saccade to the now-empty location as soon as the fixation spot was extinguished. More crucial for our current aim, two new conditions were added: When two stimuli, one on each side, had appeared at the same time, the animal was trained to make an upward saccade. On the other hand, a downward saccade was the correct response if no stimulus had appeared before the fixation target turned off. Notice that these two additional conditions forced the animal to detect, remember and explicitly report whether it had seen the visual stimuli or not, and that the direction of saccade response was disjoint from that of the visual stimuli. The discs were 1.0 degree in diameter with luminance of 1.24 cd/m^2^, unless specified otherwise in the text, against a background luminance of 0.12 cd/m^2^ (measured by a chromameter, CS-100; Minolta Photo Imaging, Mahwah, NJ). The eccentricity of the visual stimuli were adjusted to match the amplitude of saccades evoked by electrical stimulation before each muscimol injection (see below 2.4. Muscimol inactivation). However, we chose not to adjust the direction of visual stimuli, since 1) the evoked saccades were all to the upper left quadrant and the directional variation across inactivated sites was smaller than the separation between visual stimuli which was 90 degrees or more, and 2) we reasoned that the effect of inactivation would spread over time to a larger volume of tissue that included the nearest stimulus direction.

**Figure 1:**
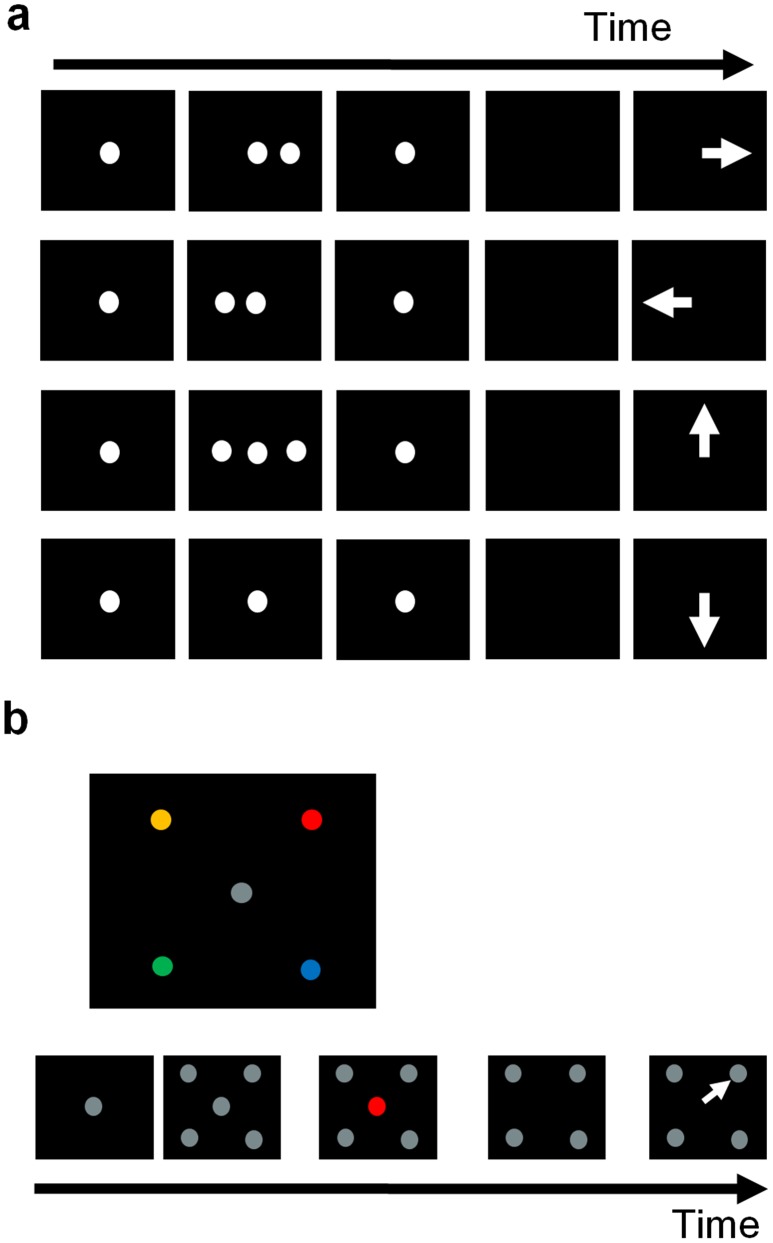
Two tasks used in the study are schematically depicted. **a**, The extended memory-guided saccade task. Visual events in task conditions are shown in rows of panels as a function of time: from the top row, two single-target conditions, bilateral stimulus condition, and no-stimulus condition. Arrows in the right-most panels indicate the correct saccade direction. **b**, The four-alternative delayed saccade task. The associations between color and spatial location shown in the top panel were pre-trained before inactivation experiments. Visual events in a trial are schematically depicted along the time line: the appearance of fixation target at the center, display of four alternative gray targets in the periphery, the onset of a color cue at the center, the color-cue turning-off signaling when to make the saccade, and the saccade to the color-matched target.

#### B) The four-alternative delayed saccade task (Figure 1b)

Upper panel shows the pre-trained location and color association, e.g. red is associated with the upper-right visual field. The association remained the same for both monkeys throughout the experiments. Lower panel depicts the events in the task: A trial began when the animal fixated at a central gray fixation disc (0.5 degree in diameter). Soon after the fixation, four gray targets appeared in the peripheral visual field, and after 400 ms the central disc changed to one of the four colors each associated with a particular target location. After a random delay ranging between 500 and 1000 ms, the central disc disappeared, which served as the cue for the animal to make a saccade response. The discs were 1.0 degree in diameter, with luminance of 1.24 cd/m^2^ against a background luminance of 0.12 cd/m^2^. The colors used for the fixation spot, peripheral targets, and central cue were equiluminant and chosen based on the CIE 1976 (L*a*b*) space, which is approximately uniform in perception of color difference [25], such that the colors were at the same distance in the space from the two neighboring ones and the gray. A chromameter (CS-100; Minolta PhotoImaging, Mahwah, NJ) was used for measuring luminance and chromaticity of the colors. The eccentricity of the peripheral targets were adjusted to match the amplitude of saccades evoked by electrical stimulation before each muscimol injection (see below 2.4. Muscimol inactivation). The direction of visual stimuli were not adjusted for the reasons stated above.

Note that, since the four gray peripheral targets were indistinguishable, a bottom-up shift of attention to the targets was not prompted by the targets nor needed for performing the task. In fact, successful performance of the task required the animal to volitionally maintain top-down attention focused on the fixation point in order to detect the color change and decide on where and when to make the saccade. The target selection was based solely on pre-learned associative memory. Here we defined attention shift in the usual sense as bottom-up visual processing of salient targets in the visual field, not disputing that the act of a saccade would entail or ensue an attentional shift.

### Experimental Procedures and Data Analysis

The performance in the task was monitored by infrared video-oculography with a sampling rate of 500 Hz (Eyelink2, SR Research Ltd, Kanata, Ontario, Canada). Saccade behavior was measured off-line using programs written in MATLAB (The Mathworks, Natick, MA, USA). Markers were available for all experimental events used in each task. The onset and offset of saccades were determined by velocity criteria (30°/s radial velocity for onset and 10°/s for offset). Correct placement of these marks was checked manually and adjusted where necessary.

Forty trials of a task was presented in a block with task conditions randomly mixed (typically, 8∼12 trials per condition). The two tasks alternated over a set of eight blocks, i.e., four blocks per task. This task alternation ensured that the inactivation effect which might change rather rapidly was monitored equally for both tasks. The block sets were run immediately before muscimol injection, and at approximately half-an-hour interval after the injection. The percent correct was assessed over the same task blocks in a block set. Error trials were classified as fixation error, motor error, and choice error, in a similar manner to our previous study [26].

### Muscimol Inactivation

Details of the inactivation methods were the same as described previously [26]. After completing the mapping of the FEF for the low-threshold region for evoking saccades, we initiated a series of experiments using muscimol injections. In each monkey, the injection sites were selected based on the following criteria: 1) Electrical stimulation at the site evoked saccades at a current level below 70 µA with a probability higher than 50% (cathode-first bipolar pulse duration 0.2 ms, pulse frequency 200 Hz, train duration 100 ms). 2) The evoked saccades were directed contralaterally, closer to the horizontal meridian than to the vertical meridian, and with the amplitude between 5 and 20 degrees of visual angle. This criterion was applied since we fixed the direction of the visual stimuli, as explained above, and the range for eccentricity adjustment was limited. For each injection site, we first lowered a microelectrode inside a guide tube. After the electrode had penetrated the dura and eye movement-related activity began to be recorded, we applied electrical stimulation at sites separated by 1 mm of electrode advancement. We located the site associated with the lowest current threshold for evoking a saccade on each penetration. The depth of this site was carefully measured using an electronic microdrive (NAN Instruments Ltd, Nazareth, Israel), and the electrode was withdrawn leaving the guide tube in place.

A 33-gauge hypodermic cannula was inserted into the guide tube and was lowered until its tip was located at the same depth previously occupied by the tip of the stimulating electrode. Injections of muscimol were made through the cannula using pressure injection from a minipump (Aladdin 1000, World Precision Instruments, Sarasota, FL, USA). The concentration of the muscimol solution was kept constant at 5 microgram/microliter. The normal volume of solution injected was 1 microliter over a period of about 2 minutes. The amount of solution injected was monitored by watching the movement of a small bubble against fiduciary marks located on the Teflon tubing connecting the pump to the injection cannula. Following the injection, the cannula was left in place for about 10 minutes and was withdrawn. Data collection began immediately after the injection and continued up to several hours in some cases. Control data were collected on the following day, and full recovery was always noted.

In each monkey, we made at least two injections of one microliter of sterile saline in each hemifield and recorded a complete set of data to control for the possibility that any negative effects produced by muscimol inactivation might have been caused by local pressure on cortical neurons or local dilution of the extracellular fluid at the injection sites.

## Results

### Visual Detection is not Affected in the Field where Saccades were Impaired by FEF Inactivation

**Figure 2:**
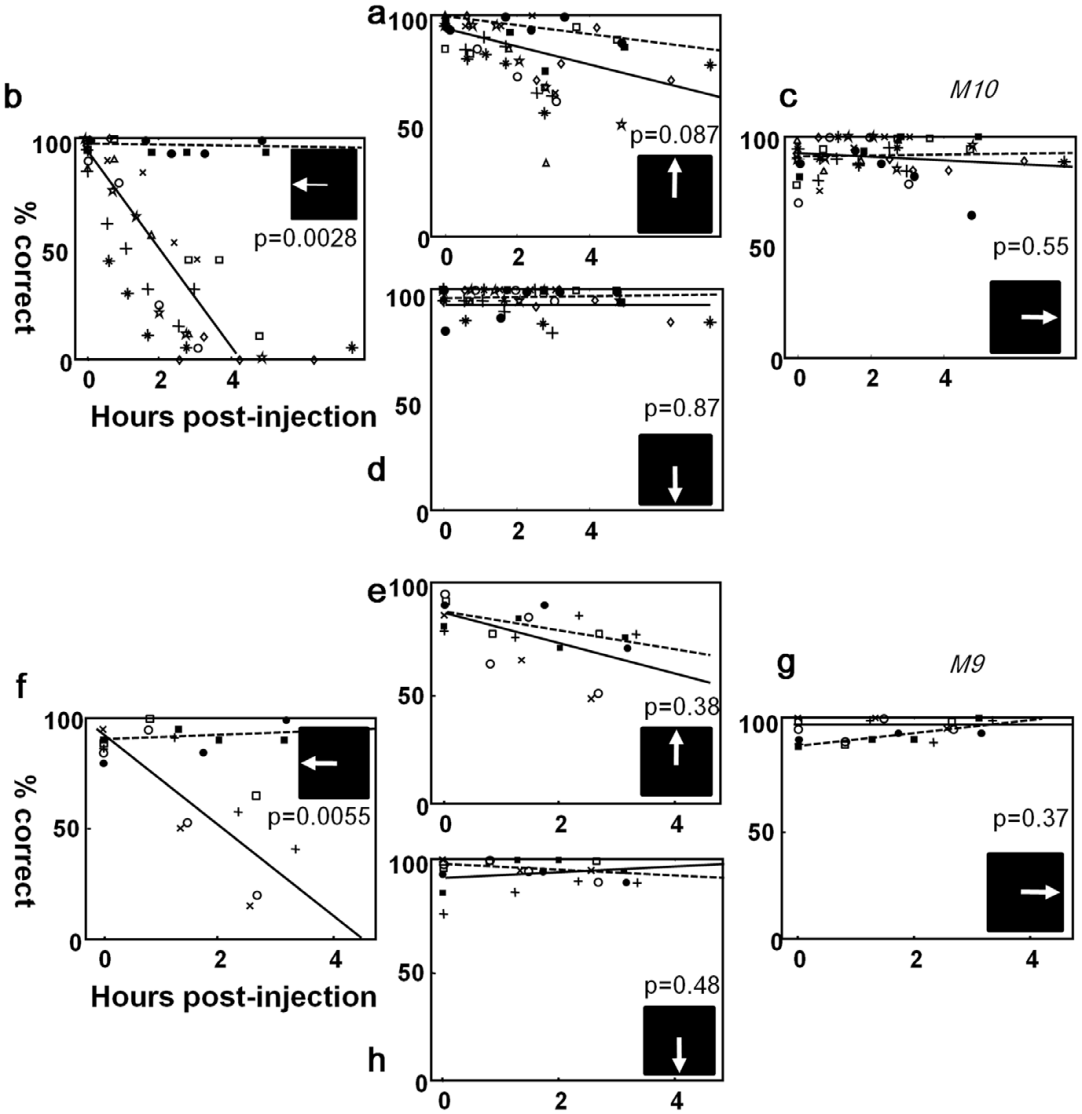
The effect of FEF inactivation on the extended memory-guided saccade task is shown. Changes in performance on the task by M10 (**a–d**) and M9 (**e–h**) are plotted as a function of time after muscimol injection into the FEF on the right side. Each data point was the percent correct out of 36 to 45 trials. Data points of a marker type were from the same inactivation session. Six inactivation experiments were performed in M10 and four in M9. Two control experiments with normal saline injection were performed in each animal with the data indicated by filled markers (circles and squares). The solid and broken lines indicate the regression over the pooled data with muscimol and saline injections, respectively. The p values are for the interaction between time-after-injection and injection type (muscimol vs. saline) in two-way ANOVA. The small icon in each panel indicate the correct saccade direction for the task condition.

The first task that we developed to dissociate the spatial confounding of visual and saccade functions was an extension of the memory-guided saccade task (Figure 1a). During FEF inactivation, the performance was severely impaired in contralateral single stimulus trials (Figure 2b,f), consistent with observations by previous investigators [18], [27]. Performance when a single target appeared in the ipsilateral field (Figure 2c,g) was nearly perfect as expected. More informatively, the inactivation had no significant impact on the bilateral- and no-stimulus conditions (Figures 2a, e and d, h, respectively). Thus, it appeared that while unable to make memory-guided saccades to contralateral visual fields, the animal showed no difficulty in monitoring visual events in the fields and reporting the brief appearance or absence of a stimulus during the delay period. These findings were consistent over six and four injection experiments in M10 and M9, respectively. Two-way analysis-of-variance test was performed on the percent correct data pooled over all injections in each monkey. The post-injection time was set as a continuous independent variable and the injection type (muscimol vs. saline) as the other discrete independent variable. The interaction between the two main effects indicated whether there was a significant difference between the injection types in the change of performance over time. The interaction was significant (p<0.05) in the case of the contralateral single stimulus condition, whereas it failed to reach significance in other conditions (p value given in each panel of Figure 2).

The direction of saccades in error trials were analyzed for further insights on the effects of FEF inactivation. The animals made error responses in a small portion of bilateral stimulus trials, which grew worse over time after muscimol or saline injections (Figures 2a, e). Most of the errors arose in target selection, rather than in saccade execution, in that the end-points were not deviated much from the target locations. The selection errors were distributed comparably between the right or left targets in both animals (two hours after muscimol injections, rightward error saccades occurred in 44.1% of total error trials and leftward ones in 39.3% in M9; 35.7% rightward and 42.7% in M10). Thus, there was no sign of propensity toward the rightward errors in this condition which would have suggested a perceptual deficit in the inactivated field. Furthermore, the proportion of rightward saccades over total error responses was not different between muscimol and saline injections: 44.1% and 35.7% for M9 and M10, respectively, after muscimol injections and 42.3% and 38.2% after saline injections (p>0.05, chi-squared test, for both animals).

With the contralateral single stimulus and two hours after muscimol injection, both monkeys made more downward errors than upward ones. Downward errors were 63.2% and 70.4% of total error trials in M9 and M10, respectively. (They never made erroneous rightward saccades in the left stimulus trials.) The downward bias of error responses might by itself be interpreted as a sign of perceptual or mnemonic disruption by FEF inactivation: That is, the animal responded as if they did not see or remember the left stimulus. However, this interpretation contradicted with the preserved performance in the bilateral condition and no rightward biasing in the error trials as stated above.

**Figure 3:**
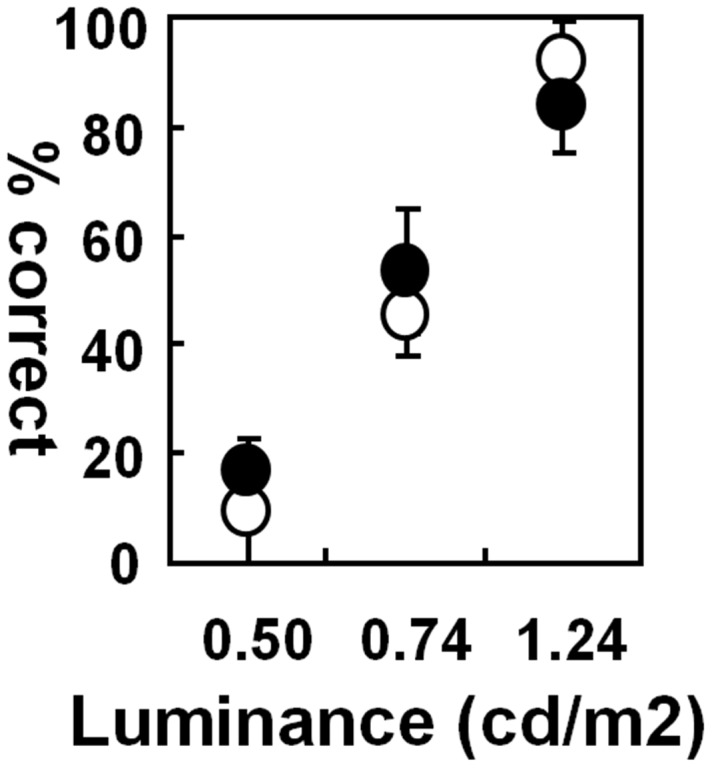
Performance in the bilateral-stimulus trials before and during FEF inactivation is plotted as a function of the luminance of the stimuli. The percent correct was calculated from a block of trials (n = 22∼25) at the luminance, administered before muscimol injections (open circles) and about two hours after them (filled circles). The mean and standard deviation (error bar) are given for the percent correct data over three muscimol injections on M10.

We therefore tested the visual threshold more directly by dimming the bilateral stimuli in three experiments with M10 (Figure 3). We reasoned that if the detection threshold were affected by the inactivation, the animal, having not perceived one stimulus, would act as if the trial were an ipsilateral single-stimulus condition, and make an incorrect, rightward saccade. The performance in the bilateral stimulus condition would then decline during the inactivation. However, the percent correct in the bilateral stimulus condition did not change significantly from what had been before inactivation. Nor did we observe changes in the direction of error saccades: When the luminance of stimuli was low, the monkey made downward saccades as if they had seen no targets at all, rather than rightward ones which would have increased if the detection threshold was elevated only in the inactivated field. No rightward saccade was observed with or without FEF inactivation at the lowest luminance level (0.5 cd/m^2^). The proportions of downward saccades in total error trials with the luminance at 0.74 cd/m^2^ were 74.3% and 76.5% before and two hours after muscimol injections, respectively, while the proportion of rightward errors were 12.8% and 10.2% in the same sessions. Evidently then, although the performance got worse at lower luminance levels, the visual threshold remained the same in both hemi-fields, before and after muscimol injection.

### The Saccade Dysfunction during FEF Inactivation is not Secondary to Dysfunction in Bottom-up Attention Shift

Given the results above that elementary visual detection was unaffected by FEF inactivation and previous reports that the FEF contributed to covert visual search [22], [28], we asked whether the inactivation primarily interfered with high-level visual processing, such as the spatial deployment of attention, and led to the impairment of memory-guided saccades as a secondary effect to the high-level visual dysfunction.

Perhaps the oculomotor function of FEF is tightly linked to and therefore conditional on its role in bottom-up shift of visual attention [9]. In other words, the impact of FEF inactivation may be specific to situations where a saccade is preceded and mediated by an exogenous attentional shift to a peripheral visual stimulus. According to this idea, it may be argued that no deficit is observed in the bilateral stimulus condition in our first task, because visual attention is not lateralized or attracted toward one side, first by the bottom-up processing of visual stimuli.

With this possibility in mind, we trained the animals on a four-alternative delayed-saccade task. In this task, a saccade target was chosen based on arbitrary learned associations between color and spatial location in the visual field (Figure 1b, the upper panel). Since the four peripheral targets were indistinguishably gray, a bottom-up shift of attention to the targets was not required for generating a saccade response. Instead, target selection was based solely on pre-learned associative memory. This task was similar to that used in our previous study [26], but was modified by a slight but important variation that dissociated visual attention and saccade intention: The animal was required to hold fixation at the central colored disc until it disappeared. This variation served two purposes: 1) the animal’s top-down attention was required to remain focused on the central disc until a saccade response was generated; 2) the determination of the correct target and the actual response, i.e., the central color cue vs. the saccade to a peripheral target, was separated in time as well as in space, reducing even further the possibility that the saccade was triggered by a bottom-up drive from the visual transients in the peripheral fields, or by a bottom-up shift of visual attention.

**Figure 4:**
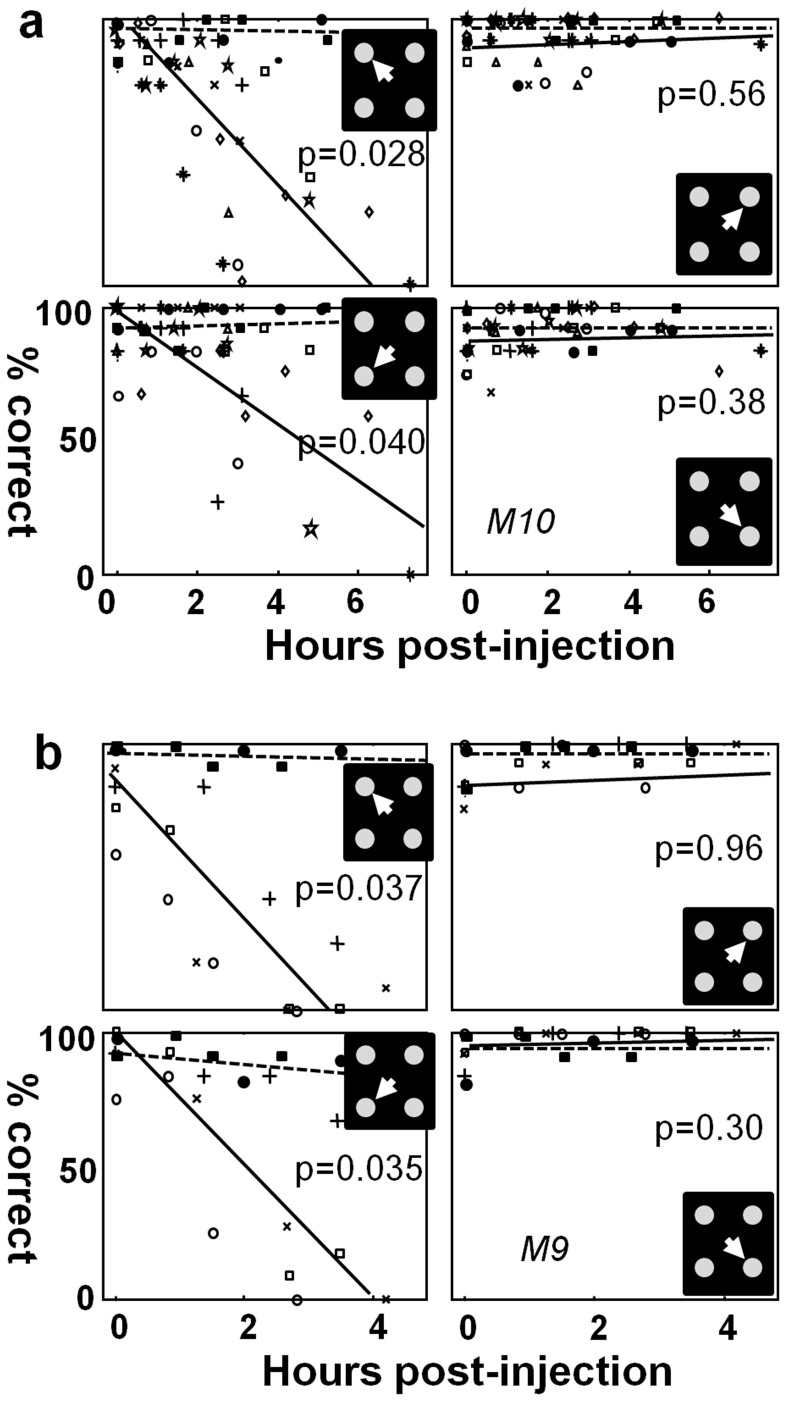
The effect of FEF inactivation on the four-alternative delayed saccade task is shown. Changes in performance on the task by two animals (M10 and M9 in **a** and **b**, respectively) are plotted as a function of time after muscimol injection into the FEF on the right side. Each data point was the percent correct out of 36 to 45 trials. Data points of a marker type were from the same inactivation session. Six inactivation experiments were performed in M10 and four in M9. Two control sessions with normal saline injection were performed in each animal with the data shown in filled circles and squares. The solid and broken lines indicate the regression over the pooled data with muscimol and saline injections, respectively. The p values are for the interaction between time-after-injection and injection type (muscimol vs. saline) in two-way ANOVA. The small icon in each panel indicate the direction of a correct saccade.

After muscimol injections to FEF, the performance on the four-alternative delayed saccade task deteriorated in a spatially specific manner (Figure 4). Errors occurred only when the correct target associated with a color cue was on the contralateral side to the injected FEF. The errors were comparable in types and proportions to those reported in our previous study [26]. Two-way analysis of variance on the percent correct data with the post-injection time and the injection type (muscimol vs. saline) revealed that the interactions between the two main effects were significant only when the correct target was in the contralateral fields (p value given in each panel of Figure 4).

## Discussion

### Transformation of Visual Input into Saccade Command by FEF

Previous studies have shown that the detrimental effects induced by surgical or reversible chemical lesions in FEF depended on task contexts [17]–[21]: The impairment is severe specifically when the saccade response is made to a remembered target. Surgical removal of FEF resulted in long-term deficits in memory-guided saccades only [20], [27], whereas muscimol inactivation produced deficits in both memory-guided and visually guided saccades, with a much worse effect on the former [18], [26]. Such task selectivity of FEF inactivation indicates that the basic processes for visual perception and saccade execution are unaffected by the lesion. On the other hand, this selectivity has often been taken as evidence for the area’s importance in high-level visual functions, such as constructing the saliency map from a visual stimulus array, or controlling visual attention based on the map [2], [5], [10], [12], [13]. A common line of thinking behind these notions is that visual input is transformed into a saccade command at the FEF, taking advantage of the co-existence of visual, visuo-movement, and movement cells with similar response fields within the same area [4].

Here, we dissected cognitive components associated with the transformation from visual input to oculomotor output, by developing two novel behavioral paradigms. Using the extended memory-guided saccade task, we spatially separated visual and motor processing, and with the four-alternative delayed saccade task, the functional linkage between bottom-up attention shift and saccadic eye movements was dissociated. Based on these cognitive dissections, we observed that the FEF inactivation did not disrupt visual detection at the affected visual fields (Figure 2). Nor was the contralateral stimulus neglected, as evidenced by the correct, up-saccade response in the bilateral stimulus condition.

The reasons remain unclear to us why the animals responded with downward saccades when a single stimulus was flashed to the left, inactivated side. This finding by itself may indicate a perceptual deficit. However, given the preserved performance with bilateral stimuli, even with low luminance stimuli, we would prefer to interpret it as a manifestation of mnemonic dysfunction either of the stimulus or of the intention to move to the left. Perhaps an instability of memory trace of the left target, in conjunction with the lack of experiencing a stimulus on the intact field might have misled the animals into judging that there had been no stimulus at all.

It did not matter for saccade impairment induced by FEF inactivation which part of the visual field received information specifying a saccade target. Saccade impairment was observed regardless of whether the information was given in the periphery as in the memory-guided saccade task, or at the center as in our four-alternative delayed saccade task. This finding suggests a rather loose functional linkage between visual and movement neurons in the FEF, contrary to the view that takes the spatial co-registration between visual and saccade response fields in the FEF neurons as evidence for a direct transformation from visual information into oculomotor commands.

### The Relationship between Attention and Oculomotor Functions by FEF

In this study, we also learned that a bottom-up attention shift was not a necessary pre-condition for FEF to generate a saccade (Figure 4). Therefore, the muscimol effect on saccade generation was independent of and likely additional to its effect on orienting bottom-up attention which was previously demonstrated using a covert visual search task [22].

As stated earlier, we do not dispute that the eye movements entail top-down attention shifting, nor that FEF plays a role in top-down or bottom-up attention control. Rather, our aim here was to examine how the role of FEF in attention control was related to that in saccade generation. Specifically, we asked whether the oculomotor aspect in FEF’s functional role depended on its role in attention, and if saccade impairments were inducible when bottom-up attention shift was not required.

It is noteworthy that the common component of the FEF inactivation effects was an inability to generate a saccade and that the disability was specific to certain task situations. The inactivation effect seemed to hinge on the requirement that saccades be created based on memorized information regardless of the memory type, be it from the short-term spatial memory stored briefly as in the memory-guided saccade task or from the long-term associative memory as in the four-alternative delayed saccade task. We speculate, in generalization of the current findings, that the FEF is specifically required in situations where saccades are generated based on internal information and in the absence of perceptually salient external stimuli specifying the target.

This notion that the FEF is critically needed for “cognitively-driven” saccades is in keeping with previous reports that demonstrated the effects on FEF activities of the feature similarity between a target and distractors [29] and feature-based attention [30]. These studies showed correlates of visual top-down selection in FEF, without the concomitant production of a saccade, indicating that the activities indeed reflected internal cognitive information which could be used for the control of top-down attention or saccade generation.

### Implications for Human Neuropsychology

Our findings demonstrate that FEF is not essential for visual detection and that the inactivation effect on saccade generation is independent of that on visual bottom-up attention. In this regard, the behavioral deficits observed in our animals resemble oculomotor apraxia in human patients in which the formation of saccade intention is considered defective.

The pattern of deficits during FEF inactivation was also consistent with hemispatial neglect, but distinct from visual extinction. In fact, a normal performance was observed in the condition with double simultaneous stimuli, ruling out the possibility that FEF lesions underlie visual extinction. This finding is consistent with human neuropsychological data which localized the neural substrates for visual extinction to more posterior parts of the cerebral cortex [31].
